# Patient–Ventilator Dyssynchrony in Critically Ill Patients

**DOI:** 10.3390/jcm10194550

**Published:** 2021-09-30

**Authors:** Bruno De Oliveira, Nahla Aljaberi, Ahmed Taha, Baraa Abduljawad, Fadi Hamed, Nadeem Rahman, Jihad Mallat

**Affiliations:** 1 Critical Care Institute, Cleveland Clinic Abu Dhabi, Al Maryah Island, Abu Dhabi P.O. Box 112412, United Arab Emirates; DeOlivB@ClevelandClinicAbuDhabi.ae (B.D.O.); AlJabeN@ClevelandClinicAbuDhabi.ae (N.A.); TahaA2@ClevelandClinicAbuDhabi.ae (A.T.); AbduljB@ClevelandClinicAbuDhabi.ae (B.A.); HamedF@ClevelandClinicAbuDhabi.ae (F.H.); RahmanN2@clevelandclinicabudhabi.ae (N.R.); 2 Cleveland Clinic Lerner College of Medicine, Case Western Reserve University, Cleveland, OH 44195, USA; 3 Faculty of Medicine, Normandy University, UNICAEN, ED 497, 1400 Caen, France; 4 Department of Anesthesiology and Critical Care Medicine, Centre Hospitalier de Lens, 62300 Lens, France

**Keywords:** assisted ventilator mode, patient–ventilator interaction, dyssynchrony, ineffective efforts, autotriggering, double triggering, flow dyssynchrony, cycling dyssynchrony, reverse triggering, airway pressure–time waveform, flow–time waveform

## Abstract

Patient–ventilator dyssynchrony is a mismatch between the patient’s respiratory efforts and mechanical ventilator delivery. Dyssynchrony can occur at any phase throughout the respiratory cycle. There are different types of dyssynchrony with different mechanisms and different potential management: trigger dyssynchrony (ineffective efforts, autotriggering, and double triggering); flow dyssynchrony, which happens during the inspiratory phase; and cycling dyssynchrony (premature cycling and delayed cycling). Dyssynchrony has been associated with patient outcomes. Thus, it is important to recognize and address these dyssynchronies at the bedside. Patient–ventilator dyssynchrony can be detected by carefully scrutinizing the airway pressure–time and flow–time waveforms displayed on the ventilator screens along with assessing the patient’s comfort. Clinicians need to know how to depict these dyssynchronies at the bedside. This review aims to define the different types of dyssynchrony and then discuss the evidence for their relationship with patient outcomes and address their potential management.

## 1. Introduction

Mechanical ventilation is the most common procedure used in the intensive care unit (ICU) [1]. Mechanical ventilatory support can be fully controlled by the ventilator and by suppressing the patient’s respiratory load (controlled ventilation) or can interact with patient breathing efforts (assisted/supported ventilation), depending on the severity and timing of the insult [2]. A perfect mechanical ventilation delivery would diminish respiratory distress by minimizing excessive respiratory load while preserving a suitable level of spontaneous effort (limiting diaphragm muscle atrophy) with appropriate patient–ventilator interaction. However, this balance is fragile, and patient–ventilator dyssynchrony occurs when this situation is not fulfilled.

### 1.1. What Is Patient–Ventilator Dyssynchrony?

In positive pressure ventilation, the clinician faces two possibilities: either the machine controls all phases of ventilation, or there are interactions between the ventilator and the patient who remains able to develop respiratory efforts [3].

In the first case, the patient is usually sedated and in some cases paralyzed with neuromuscular blockers. This strategy could have deleterious effects, namely delirium [4], weakness [5], and prolonged ICU length of stay [6]. 

In the second case, the clinician would recognize and manage noneffective and possibly harmful interactions between the ventilator and patient breaths. This inappropriate interaction is called patient–ventilator dyssynchrony. Thus, dyssynchrony is defined as a mismatch between the patients’ own inspiratory and expiratory times and the mechanical ventilator delivery times [7]. This mismatch usually unveils a discrepancy between the patients’ requirements and the machine’s amount of support. Dyssynchrony detection needs a rigorous examination of flow and airway pressure waveforms displayed on the ventilator screen [8], together with a clinical examination of the patient’s breathing pattern. Dyssynchronies can easily be missed by clinicians who may not recognize them.

### 1.2. Why Should We Care about Patient–Ventilator Dyssynchrony?

It is approximately estimated that at least one-third of patients manifest frequent dyssynchrony during mechanical ventilation [9,10,11,12]. Dyssynchronies, among other effects, overload respiratory muscles, increase alveolar distension with the risk of lung injury, and compromise sleep quality resulting in more sedation and delirium. Indeed, observational studies have consistently found dyssynchrony to be associated with poor outcomes, such as longer duration of ventilatory support and higher mortality [10,13]. 

Decreasing dyssynchrony by adjusting ventilatory settings is often doable [14,15]. An adequately synchronized interaction between patient and ventilator is desirable due to lower sedation requirements; progressive improvement in respiratory muscle function; and, consequently, shortened ventilation time, fewer complications, and earlier liberation from mechanical ventilation [16].

This review aims to define the different types of dyssynchrony and then discuss the evidence for their relationship with patient outcomes and address their potential management. Only dyssynchronies in adult patients during invasive mechanical ventilation will be reviewed.

## 2. Classification of Patient–Ventilator Dyssynchrony

There are multiple ways to classify patient–ventilator dyssynchrony as one can take different approaches to the problem: either considering foremost the primary condition that leads to the mismatch between patient and mechanical systems or considering first the phase of the respiratory cycle when they occur. 

For the first classification, the two main groups are dyssynchronies arising from insufficient assistance in high respiratory drive patients and overassistance in low-respiratory-drive patients [17]. The respiratory phase classification includes trigger dyssynchrony, flow dyssynchrony, and cycling dyssynchrony [18]. In this review, we describe only the dyssynchrony by respiratory phases.

## 3. Dyssynchrony by Respiratory Phases

### 3.1. Dyssynchrony of the Trigger Phase

Forms of trigger dyssynchrony discussed here are delayed triggering/ineffective efforts and autotriggering. 

#### 3.1.1. Delayed Triggering and Ineffective Efforts

When a patient triggers the ventilator, the patient increases intrathoracic volume, which results in a drop in airway pressure and a rise in airflow. If the pressure (called pressure triggering) or airflow (called flow triggering) reaches the threshold set by the clinician, then the ventilator delivers a breath.

There are cases where a patient wants (or attempts) to start a breath and trigger the ventilator but this fails. This can relate to the ventilator’s inability to detect the patient’s effort and start the inspiratory phase of the mechanically delivered breath. This is known as ineffective efforts. Ineffective efforts are the most common type of dyssynchrony in patients under invasive mechanical ventilation, both early in the course of the disease and during prolonged mechanical ventilation. A quarter to 38% of invasive mechanically ventilated patients experience ineffective triggering [10,11,13,19]. Their incidence is higher in patients with chronic obstructive lung disease (COPD) [14].

**Figure 1:**
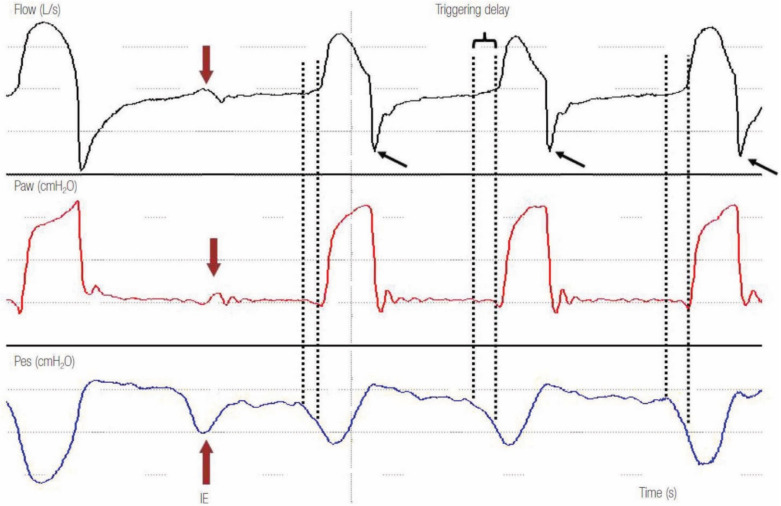
Example of a tracing with ineffective efforts and delayed triggering in pressure support ventilation mode. Note that the second decrease in esophageal pressure (Pes) (red arrow), which represents ineffective effort (IE) of the patient, is not followed by a ventilator breath. At the time of the missed effort, a positive deflection in the airway flow waveform (black arrow) and a negative deflection in the airway pressure (Paw) (red arrow) can be noted. Observe that in every ventilator breath, there is a time gap (delayed triggering) between the start of neural inspiration (first dotted line) and the start of ventilator inspiration (second dotted line). Notice the spike early in expiratory flow (black arrows) after each breath that suggests high airway resistance causing incomplete exhalation and delayed triggering [18].

Clinical evidence of inspiratory efforts by the expansion of the patient’s thoracic volume (that may be subtle, such as a small retraction above the suprasternal notch) without accompanying mechanical insufflation is specific for the diagnosis [9] but not very sensitive. The gold standard for their recognition is a negative deflection in esophageal pressure (Pes) or a significant increase in the electrical activity of the diaphragm (EAdi) not followed by a mechanical breath (Figure 1) [8]. However, both methods do not represent the standard of clinical practice and need the insertion of specific catheters. In most cases, ineffective efforts can be recognized noninvasively by scrutinizing the flow–time and airway pressure–time waveforms on the ventilator screen. Ineffective triggering is manifested by a decrease in airway pressure waveform along with an increase in airflow tracing during expiration (Figure 1) [8,9,16,17,18,19]. Triggering delay is the time interval between the start of the neural and mechanical inspiration [11,18]. If Pes or EAdi monitoring is available, it is recognized as the time elapsed between the decrease in Pes or rises in EAdi (commencement of neural inspiration) and the sudden increase of airflow or airway pressure (beginning of mechanical inspiration) (Figure 1) [8].

Ineffective efforts and delayed triggering can be due to ventilator settings and/or patients’ issues [10,14,20,21]. Indeed, low triggering sensitivity and delayed opening of the expiratory valve result in ineffective or delayed triggering. These factors are set up by the clinician. The most important cause of ineffective efforts and delayed triggering related to patient disease (COPD) is the presence of dynamic hyperinflation that generates intrinsic positive end-expiratory pressure (PEPEi). This happens because the patient’s ventilatory muscles must first counterbalance the PEEPi in the alveoli before the ventilator senses any variation in flow or pressure and then triggers the next breath: this is the “threshold load” [22,23,24,25]. This produces a delay between the start of inspiratory effort and ventilator triggering, and in severe cases of dynamic hyperinflation, the patient might fail to trigger the ventilator (ineffective triggering). This can be recognized by noticing abrupt expiratory flow end before a triggered breath (Figure 1). A spike in expiratory flow can also be appreciated at the early expiratory phase, suggesting increased airway resistance (Figure 1). The ineffective efforts are usually present in COPD patients with a very high respiratory compliance (>88 mL/CmH_2_O) [26].

Patients with a low respiratory drive (sedation, central nervous diseases, etc.) and weak inspiratory muscles (critical illness polyneuromyopathy, myasthenia, etc.) can also have ineffective respiratory efforts that do not translate to mechanically delivered efforts. This is suggested by a decrease in pressure and an increase in airway flow that is not followed by a typical breath.

#### 3.1.2. Autotriggering

There are situations where the patient makes no attempt at breathing, and the ventilator will trigger a breath on its own. This is called autotriggering [27,28] and is caused by a sensed change in pressure or flow by the ventilator that is unrelated to the patient’s efforts, namely in cases of leaks in the patient–ventilator circuit at any level or very sensitive trigger settings that will activate following thoracic pressure fluctuations derived from the cardiac cycle. A leak will cause autotriggering in one of two ways: (1) for flow-triggered mechanical breaths, the trigger is a sensed decrease in airflow in the expiratory limb of the ventilator circuit, and (2) for pressure-triggered breaths, the trigger is a sufficient drop in pressure caused by the leak. In the case of a very sensitive flow-trigger, the cardiac pacing transmitted through the tracheal wall could be misunderstood as the patient’s inspiratory efforts and trigger mechanical breaths. 

**Figure 2:**
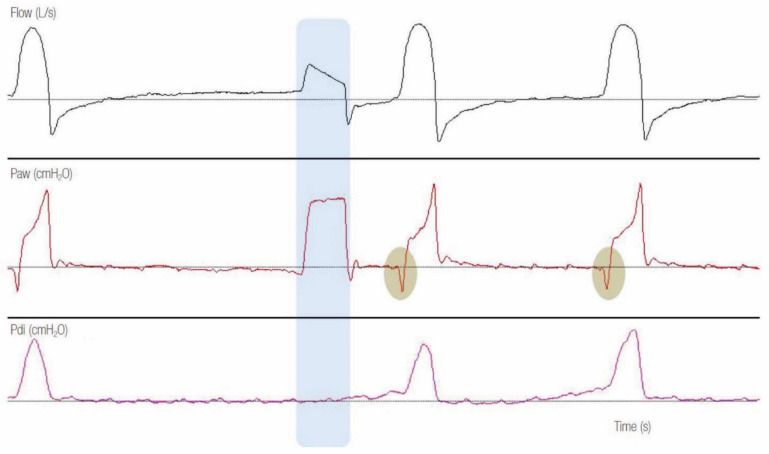
Example of a tracing with autotriggering in pressure support ventilation mode. Note the absence of transdiaphragmatic pressure (Pdi) increase, indicating no inspiratory effort, before the second ventilator breath (blue shaded area). Observe that, in comparison to patient-triggered breaths, where a decrease in airway pressure (Paw) is noticed before the start of ventilator inflation (grey shaded areas), there is no twisting in the Paw (no decrease in Paw) in the autotriggered breath [18].

The lack of a drop in Pes or airway pressure before the delivery phase, particularly in the presence of a zero flow long enough before the mechanical breath, indicates that the breath is not triggered by the patient (Figure 2). 

### 3.2. Dyssynchrony of the Flow Phase

**Figure 3:**
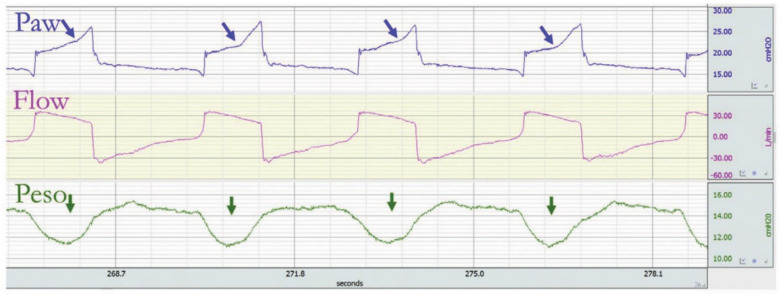
Example of a tracing with relative flow starvation. The ventilator is in a volume assist–control mode with decelerating flow. The flow delivered by the ventilator does not meet the patient’s need. During the inspiration phase, there is a concave deflection of the airway pressure (Paw) (blue arrow), whereas the esophageal pressure (Peso) displays a negative deflection indicating patient’s effort (green arrow). Reprinted with permission from [17].

Insufficient flow delivery results in patient discomfort and increased inspiratory effort [19,29,30]. This effort from the patient produces a decrease in the pressure–time ventilator curve. The insufficient flow delivery commonly occurs in flow-targeted volume assist–control ventilation if the inspiratory flow is not correctly set, being too low for the patient’s respiratory need [31,32]. In volume-controlled breaths, the inspiratory flow cannot be increased by the patient’s efforts, and as this effort increases, the airway pressure consequently falls, giving the pressure–time curve the characteristic “scooped-out” look that may be severe enough to decrease peak airway pressure [10,33] (Figure 3). Marini et al. were the first to report relative flow hunger and to raise the significance of appropriately setting inspiratory flow during assist–control ventilation [29].

### 3.3. Dyssynchrony of the Cycling Phase

The ventilator cycles or stops the flow to terminate the mechanical inspiratory phase and begin mechanical expiration based on different criteria depending on mode settings. Specifically, in flow-targeted modes, delivered tidal volume and cycle time are set by the physician and cannot change with patients’ efforts. In pressure-targeted modes, the cycling criteria are either a set inspiratory time (T_I_) in pressure assist–control ventilation or a flow cycle setting in pressure support ventilation.

Cycling dyssynchrony happens when the neural T_I_ and the ventilator T_I_ are mismatched [21,34].

#### 3.3.1. Premature Cycling Off

Short cycling dyssynchrony occurs when the mechanical T_I_ is shorter than the neural T_I_. This can leave the patient uncomfortable (air hungry) as activation of inspiratory muscles during mechanical expiration (lengthening) results in an eccentric contraction of the diaphragm, which is potentially injurious for the respiratory muscles [35,36]. When the inspiratory muscle contraction is strong enough, it can overcome the respiratory system’s elastic recoil pressure and trigger a second mechanical breath, known as double triggering, before full deflation of the first one, leading to an increased total tidal volume. This phenomenon is also named breath stacking [37] because of the occurrence of a new breath on top of the previous one. Breath stacking is common during low-tidal-volume ventilation in patients with acute respiratory distress syndrome not receiving neuromuscular blocking agents [37]. Double triggering was one of the most common ventilator dyssynchronies, and it seems to increase in incidence in patients with worse lung injury and high respiratory drive [8].

Premature cycling was associated with particular ventilator settings, such as a set of low tidal volume [37], a shorter set of inflation time [38], and being ventilated with assist–control ventilation [10]. 

Premature cycling off can result in an “inverse ratio” ventilation with the risk of increasing mean airway pressure. This potential complication can be very harmful in ARDS patients, aggravating lung injury. Switching to pressure support may better reduce the frequency of double triggering than increasing sedatives and analgesia [39].

**Figure 4:**
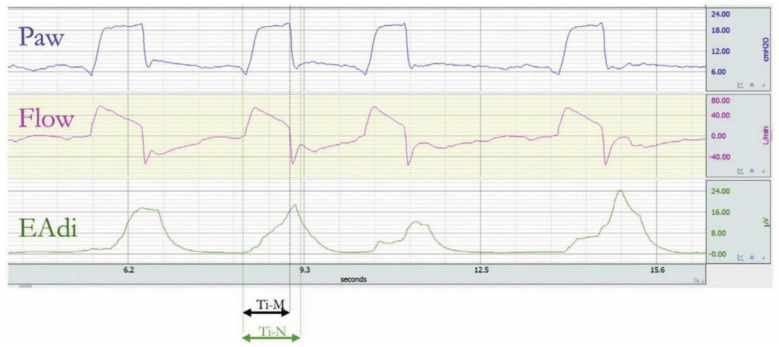
Example of premature cycling in pressure support ventilation. The patient’s neural inspiration time (Ti-N), which is from triggering to the moment the electrical activity of the diaphragm (EAdi) drops to 70% of its maximal value, is longer than the breath delivered by the ventilator (Ti-M). Reprinted with permission from [17].

Premature cycling off can be appreciated on the ventilator screen by observing, in the flow–time curve, an abrupt drop from the peak expiratory flow, which lasts few milliseconds, followed by an increase and then a decrease gradually to zero toward the end of expiration (Figure 4) [18].

#### 3.3.2. Delayed Cycling Off

Dyssynchrony in this phase is usually related to a longer ventilator T_I_ set than patient neural T_I_, which means mechanical insufflation continues after neural inspiration has stopped or even during neural expiration. This can lead to an increase in work of breathing as the patient is actively using his or her expiratory muscles, struggling to terminate the mechanical inspiration. Additionally, excessive ventilator T_I_ results in a larger tidal volume that can lead to overdistention that may further shorten the neural T_I_ [40], contributing more to the mismatch between a long mechanical insufflation and a short neural T_I_. This can be particularly problematic in COPD or asthma patients ventilated with pressure support ventilation. Under these circumstances, the increased airway resistance causes the delivered inspiratory airflow to decrease slowly, which results in insufficient expiratory time since during pressure support ventilation, the ventilator switches to expiration when flow declines to a set percentage of peak inspiratory flow. These effects may contribute to worsening dynamic hyperinflation (higher PEEPi) [41] and consequent trigger dyssynchronies (ineffective triggering, delayed triggering). 

**Figure 5:**
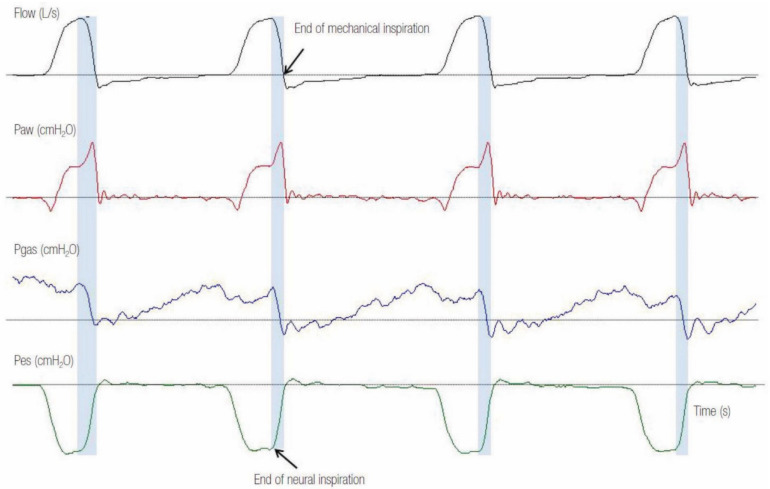
Example of delayed cycling in pressure support ventilation. There is a significant time delay (blue shaded area) between the end of neural inspiration, depicted by a rapid increase in esophageal pressure (Pes), and the end of mechanical inspiration, indicated by the termination of inspiratory flow (inspiratory flow equals zero). Notice the rapid upsurge of Paw near the end of mechanical inspiration, indicating inspiratory muscle relaxation [18].

Delayed cycling can be recognized by an abrupt spike of the airway pressure near the end of mechanical inspiration on the pressure–time waveform (Figure 5). High pressure support and low rise time are predisposing factors for this asynchrony [42]. High tidal volume, increased T_I_, low insufflation airflow, and inspiratory pause may result in delayed cycling in assisted volume control mode [42].

### 3.4. Reverse Triggering

**Figure 6:**
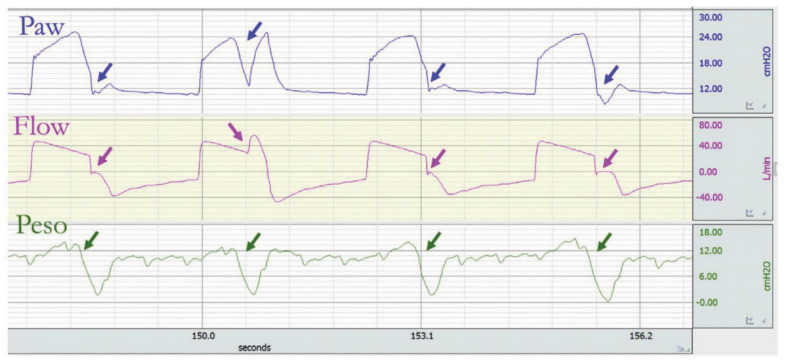
Reverse triggering in volume assist–control with decelerating flow. Each insufflation is triggered by the ventilator; in the middle (breath 2) or at the end of the inspiration phase (breaths 1, 3, and 4), a negative deflection of the esophageal pressure (Peso) happens (green arrow), leading to a negative deflection of the airway pressure (Paw) (blue arrow) and increasing flow during the ventilator insufflation phase or reducing it during the ventilator expiratory phase (pink arrows). Reprinted with permission from [17].

**Figure 7:**
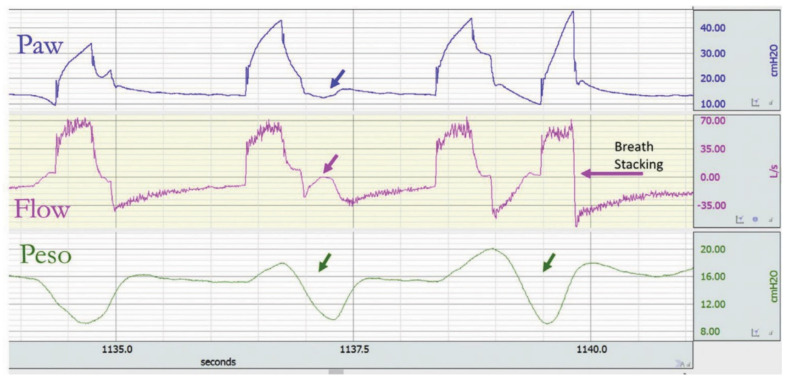
Reverse triggering and breath stacking in volume assist–control. The first breath is patient-triggered; the second is triggered by the ventilator and followed by a reverse triggering (first green arrow) starting in the late phase of ventilator insufflation and continuing during the early phase of ventilator exsufflation leading to a positive deflection of the flow (pink arrow) not triggering a new breath; and the third breath is ventilator-triggered, followed by a reverse triggering (second green arrow). This reverse triggering leads to a fourth breath before complete exhalation of the previous one (breath stacking). Reprinted with permission from [17].

In heavily sedated patients, the mechanical breath delivered by the ventilator can trigger a muscular effort. This is called reverse triggering, first described by Akoumianaki et al. [43], who observed, in deeply sedated patients under controlled mechanical ventilation, the occurrence of patient inspiratory efforts (inspiratory activity of the respiratory muscles) that were triggered by the ventilator insufflations. In the absence of Pes or EAdi monitoring, a rise in expiratory flow or decline in inspiratory airway pressure that occurs later in the respiratory cycle can point to this asynchrony (Figure 6). Furthermore, if the inspiratory effort is sufficiently strong, the ventilator can generate a second breath, resulting in breath stacking (Figure 7) like in patients with a high respiratory drive. However, the difference with double triggering induced by a high drive is that the first breath in the case of reverse triggering is not triggered by patients.

The mechanism of this phenomenon is unclear but somehow related to respiratory entrainment (fixed, repetitive, and temporal relationship between mechanical breath and inspiratory effort) previously described in healthy humans [44,45] and animals [46]. This phenomenon does not seem to be mediated by vagal output as initially thought, since it was described in patients after lung transplant [45] and brain-dead patients [47]. 

## 4. Consequences of Dyssynchrony

It is hard to establish the true prevalence of dyssynchronies mainly due to different definitions and criteria, methods of detection, ventilatory modes, and duration and timing of observation [10,20,25,26,48]. In any case, triggering dyssynchrony is by far the most well studied, and delayed/missed triggering dyssynchronies are the most commonly reported in 26 to 82% of mechanically ventilated patients depending on the studied population, ventilator settings, and measurement methods used [26]. As expected, trigger dyssynchronies were more prevalent in patients with COPD and asthma that are more prone to develop dynamic hyperinflation [20,26]. However, trigger dyssynchronies can go undetected in about 20% without the use of invasive monitoring tools such as Pes and EAdi [26]. The incidence of other types of dyssynchrony (double triggering, flow dyssynchrony, and cycle dyssynchrony) has not been as well investigated, with a reported incidence of about 10% [26,37,49]. However, the reported incidence is likely underestimated.

Experienced physicians in the ICU would probably agree that every mechanically ventilated patient would present at some point some minor form of patient–ventilator dyssynchrony, and it must be emphasized that dyssynchrony is not always benign. Indeed, many reports suggest an association between dyssynchrony and poor outcomes, including higher mortality and increased duration of mechanical ventilation and ICU length of stay [9,10,13,39,48]. Ineffective efforts were shown to be associated with a longer mechanical ventilation duration and a lower rate of weaning success [9]. Thille et al., in awake patients under assist–control ventilation or pressure support ventilation modes, demonstrated that dyssynchrony index (ratio defined by the number of dyssynchronous breaths over the total number of breaths) greater than 10% was associated with increased duration of mechanical ventilation and increased rate of tracheostomy [10]. Other authors confirmed the link between dyssynchrony index ≥ 10 and outcomes (longer durations of mechanical ventilation, ICU, and hospital length of stay) [13,48]. Dyssynchronous breathing has been shown to result in episodic increases in transpulmonary pressure, possibly causing ventilation-associated lung injury [50]. Whether the association between adverse outcomes and patient–ventilator dyssynchrony implies causation or only depicts a common association of a poor prognosis remains unclear.

## 5. How to Improve Synchrony

### 5.1. Trigger Synchrony

Both cases of untriggered efforts and extra triggering can be managed in most situations at the bedside.

Firstly, the clinician must be aware of the mode and setting of the ventilator’s trigger sensor: either pressure, flow, or both. The trigger sensitivity requires individual patient adjustment and should be set at a level at which all patient inspiratory efforts translate into a cycle start.

In cases of untriggered efforts, special attention should be brought to the possibility of intrinsic PEEP (PEEPi) development and an increasing pressure gradient that the patient’s efforts fail to overcome. The first step should be to try and minimize PEEPi (usually by decreasing minute ventilation or the global need for increased ventilation that is driving the patient’s respiratory drive). The second step would be to reduce the triggering load by approximating the circuit PEEP to the total PEEPi to a level of about 75% of the total PEEPi. This should result in reduced work of breathing, increases in triggered breaths, and also increased patient comfort [9,22,23]. However, excessive applied PEEP above the PEEPi will either increase the end-inspiratory pressure in volume-controlled ventilation mode or reduce the generated tidal volume in pressure-targeted ventilation. As long as the external PEEP is lower than the PEEPi, the relationship between inspiratory pressure and tidal volume would not change [51,52]. Thille et al. showed that the most effective way to reduce ineffective efforts was to decrease overassistance by reducing the pressure support level in patients ventilated with pressure support ventilation mode [14]. Adjusting T_I_ to allow smaller tidal volume was also an effective method to reduce ineffective efforts [53].

For autotriggering, one should evaluate possible reversible causes such as leaks or water in the circuit and/or increase the trigger sensitivity level [54]. 

### 5.2. Flow Synchrony

Firstly, in the case of using flow-targeted breaths, the flow cannot vary with patients’ efforts and can lead to synchrony issues. This can be somewhat mitigated by adjusting the flow and its shape (sinusoidal, square, decelerating) [55]. 

Pressure-targeted modes offer advantages in this regard since the set pressure target will allow for the ventilator to variate the flow until a certain pressure is obtained. This has been shown to increase synchrony [31,32,51,55]. Moreover, the slope (pressure rise time) can be adjusted, and patients producing higher degrees of effort will synchronize better using low rise times [56,57].

### 5.3. Cycling Synchrony

Attaining breath cycling synchrony requires delivering an adequate tidal volume in accordance with patient requirements and balancing neural and ventilator T_I_. 

Premature cycling can be addressed by increasing machine T_I_ or inspiratory flow or applying end-inspiratory pause in control modes [8,33]. A higher support level, slower rising time, and lower flow threshold for cycling can help to minimize premature dyssynchrony in pressure support ventilation mode [8,57]. When increasing volume is not an option, usually in cases where strict low-volume protective ventilation is required, a higher level of sedation will be necessary.

Delayed cycling can be minimized by decreasing the machine T_I_, increasing the flow threshold of cycling off, decreasing the support level, and increasing the rise pressure time in case of pressure support ventilation. 

## 6. Newer Ventilator Modes and Patient–Ventilator Synchrony

### 6.1. Neurally Adjusted Ventilatory Assist (NAVA)

NAVA requires inserting a special esophageal catheter with an array of electrodes that sense EAdi [58]. These sensors detect the start, strength, and end of inspiratory efforts directly. In this mode, the ventilator breath is triggered and cycled off by the EAdi signal. The ventilator assistance can vary with the esophageal–diaphragmatic signal’s amplitude detected according to a physician’s preset gain. 

Several clinical studies with NAVA showed a significant reduction in the number of asynchronies in patients when compared to conventional modes [12,59,60]. The obvious caveat is that this mode requires an intact respiratory drive of the patient. Thus, NAVA must be used cautiously in patients with unpredictable ventilatory drives from either disease or drugs. Moreover, in patients with high respiratory drive and extremely low respiratory compliance, premature cycling and double triggering can still occur [61].

### 6.2. Proportional Assist Ventilation (PAV)

In PAV, the ventilator regularly estimates the patient’s muscular effort based on measured airway pressure, lung compliance, flow, and resistance to determine the pressure of each individual breath in proportion to the desired reduction in the patient’s work of breathing [62]. The physician presets the level (as a percentage) of ventilator assistance in proportion to the patient’s muscular effort. Contrary to NAVA, PAV has no effects on triggering of the breath but improves patient–ventilator synchrony during inspiration by matching the patient’s inspiratory demand and cycling since the ventilator cycles breath when sensed flow demand has stopped. Most clinical studies showed that patients on PAV have less occurrence of ineffective triggering and other dyssynchronies than patients on pressure support ventilation [63,64]. However, data regarding the association between PAV and outcomes (duration of mechanical ventilation, sedation requirements, ICU length of stay, and mortality) are lacking. Moreover, it is not obvious what the ideal PAV adjustments (gain) should be in different clinical situations. As with NAVA, there is no minimum pressure or flow supplied; thus, PAV must be used cautiously in patients with unpredictable ventilatory drives from either disease or drugs.

## 7. Conclusions

Assisted mechanical ventilation modes offer significant advantages over controlled modes in terms of ventilator-induced diaphragmatic dysfunction and sedation needs. However, patient–ventilator dyssynchrony is a common and polymorphic event that may happen at any phase throughout the respiratory cycle. Various types of asynchronies have diverse physiologic causes and need different management. Dyssynchrony inflicts high loads on respiratory muscles, inducing muscle fatigue. An association between dyssynchrony and outcomes was found in observational studies; however, it is not clear if this link was confounded by the patients’ severity or it was a causal association. Moreover, it is not clear if all dyssynchronies are harmful and if reducing dyssynchrony would result in improving patient outcomes. Clinicians must know how to depict patient–ventilator dyssynchronies by understanding airway pressure and flow waveforms. Physicians should also be able to uncover the relationship between dyssynchronies and ventilation modalities (flow-triggered, pressure-triggered, assist–control ventilation) and determine their clinical impacts and rates.

## Data Availability

Not applicable.

## Abbreviations

Pes, esophageal pressure; EAdi, electrical activity of the diaphragm; PEEPi. intrinsic positive end-expiratory pressure; NAVA, neurally adjusted ventilatory assist; PAV, proportional assist ventilation; T_I_, inspiratory time.
